# Problems, Causes, Impact, and Preventive Measures for Addressing Violence Faced by Nurses in Healthcare Settings in Thailand

**DOI:** 10.3390/ijerph23081018

**Published:** 2026-08-04

**Authors:** Nithinan Mahawan, Rachadaporn Jantasuwan, Hasanah Pairoh

**Affiliations:** 1 Division of Community Nursing, School of Nursing, The Excellence Center of Community Health Promotion, Walailak University, Nakhon Si Thammarat 80161, Thailand; nithinan.ma@wu.ac.th; 2 Department of Professional Nursing Studies, Kulliyyah of Nursing, International Islamic University Malaysia, Kuantan 25200, Pahang, Malaysia; hasanahpairoh@iium.edu.my

**Keywords:** workplace violence, psychological violence, violence exposure, health effect

## Abstract

**Highlights:**

**Public health relevance—How does this work relate to a public health issue?**

- Workplace violence (WPV) is a significant occupational and public health problem that threatens nurses’ safety, well-being, workforce retention, and quality of patient care.
- Understanding the prevalence, causes, and consequences of WPV is essential for strengthening healthcare systems and creating safer work environments for healthcare professionals.

**Public health significance—Why is this work of significance to public health?**

- WPV affected nearly half of nurses in this study and was associated with substantial psychological distress, reduced professional confidence, and intentions to leave the profession, highlighting its role as a critical threat to healthcare workforce sustainability. The study provides evidence on the prevalence, causes, and consequences of WPV among nurses in Thailand, supporting the development of evidence-based policies and organizational interventions to create safer healthcare environments.

**Public health implications—What are the key implications or messages for practitioners, policy makers and/or researchers in public health?**

- Healthcare organizations should implement targeted prevention strategies for high-risk settings, including medical wards and emergency departments, while strengthening incident reporting systems, security measures, and staff support programs.
- Policymakers and healthcare leaders should prioritize workforce protection policies, adequate staffing, violence prevention training, and organizational interventions to improve nurse retention, workforce resilience, and patient safety.

**Abstract:**

Workplace violence (WPV) is a major occupational hazard for nurses in Thailand, negatively affecting psychological well-being, professional confidence, and patient care. In this quantitative cross-sectional analytic study involving 415 nurses, 43.61% reported experiencing at least one incident of WPV during the previous 12 months. Verbal abuse (41.20%) and physical violence (13.25%) were the most frequently reported forms, with a higher prevalence observed in urban hospitals than in rural hospitals (53.66% vs. 33.81%). Higher WPV risk was associated with working in medical wards (adjusted incidence rate ratio [Adj IRR] 3.33) and emergency departments (Adj IRR 2.47), caring for male-only patients (Adj IRR 1.87), younger age (Adj IRR 3.31), divorced or separated status (Adj IRR 3.05), rotating day and night shifts (Adj IRR 2.03), and extreme concern about WPV (Adj IRR 4.57). The psychological impact was considerable. Hypervigilance was the most common response (66.30%), especially following physical violence (76.36%). More than 41% of affected nurses reported intentions to resign or transfer and reduced professional confidence. Reporting incidents was often perceived as ineffective, contributing to underreporting. Nurses identified clear disciplinary measures, increased staffing, improved workplace environments, stronger security systems, and organizational support as key prevention priorities. These findings highlight the urgent need for context-specific interventions, targeted staff training, and organizational strategies to protect nurse well-being, strengthen workforce resilience, and maintain quality patient care.

## 1. Introduction

Workplace violence (WPV) in healthcare is an increasing global concern, especially among nurses due to their frontline roles and frequent interactions with patients, families, and colleagues [1,2]. Such exposure increases vulnerability to various forms of WPV, including physical assault, verbal abuse, and sexual harassment [2,3]. These experiences detrimentally impact physical and psychological well-being, causing stress, burnout, job dissatisfaction, and turnover intention, which ultimately jeopardize patient care quality and continuity [4,5,6,7].

WPV against nurses is very common in Thailand. Approximately half to two-thirds of nurses experience at least one incident annually [8,9]. In Bangkok, WPV against nurses is a significant concern. Within the past year, 60% of nurses reported experiencing psychological abuse in the workplace, while 9.1% experienced physical WPV [10]. The burden of WPV is particularly pronounced in high-pressure settings, such as emergency departments, where as many as 88.4% of healthcare workers have been subjected to WPV [11]. Many factors underlie WPV; however, in Thailand, it is mostly driven by structural challenges, such as an uneven workforce and a growing demand for services. An older population, the increasing prevalence of chronic diseases, and more people receiving healthcare through the Universal Health Coverage plan all contribute to these stresses [12,13].

WPV undermines well-being, increases burnout, lowers job satisfaction, and raises intentions to resign among Thai nurses, compromising workforce stability and patient care [11,14]. These effects further destabilize a workforce that is already insufficient and burdened with large workloads. WPV also indirectly increases reliance on overtime and temporary labor while diminishing service capacity [13]. Ultimately, WPV affects the quality and continuity of care, complicating the treatment of various diseases. This is particularly critical in Thailand, which is among the top 30 countries with the highest tuberculosis (TB) and HIV burden [15], where effective disease control relies heavily on sustained, high-quality nursing care, treatment adherence, and strict infection control practices. Disruptions in staffing and care continuity increase the risk of delayed diagnosis, treatment interruption, and onward transmission of TB to surrounding communities [16,17,18]. Furthermore, the reporting and documentation of WPV incidents are often incomplete because of multiple nursing, management, and organizational barriers. Nurses’ fear of negative consequences, limited knowledge of reporting procedures, lack of visible organizational responses, unsupportive reporting cultures, and inadequate reporting policies and systems may contribute to underreporting. Consequently, incomplete reporting may compromise the accuracy of surveillance data and weaken organizational capacity to monitor, prevent, and respond effectively to WPV [19].

Despite increasing concern about WPV, relevant studies in Thailand remain limited. Most studies have focused on single settings, particularly urban tertiary hospitals, with little attention given to variations across different healthcare contexts [8,11]. Consequently, our comprehension of the distinctions in WPV between urban and rural environments remains inadequate, as disparities in resources, personnel, and response systems would influence both the incidence and management of WPV [10,20]. These differences can affect how nurses experience WPV, its impact, and the type of support needed. Urban hospitals may require more effective procedures, stricter security, and better workload management, while rural areas may require solutions for staffing shortages, limited support, and reporting gaps. This study investigated the problems, causes, impacts, and preventive measures related to WPV against nurses in Thailand and compared urban and rural settings in these respects. Specifically, the study aimed to:

1. Examine the prevalence and patterns of WPV in urban and rural hospitals.
2. Identify demographic, work-related, and organizational factors associated with WPV.
3. Assess the psychological and occupational outcomes associated with WPV.
4. Explore nurses’ perspectives on effective, context-specific strategies for preventing WPV.

## 2. Materials and Methods

### 2.1. Study Sample and Procedure

This quantitative cross-sectional analytic study was conducted between March and October 2025. Ethical approval was obtained from the Human Research Ethics Committee of Walailak University (Approval No. WUEC-25-007-02). Additional ethical approvals were granted by the participating urban hospitals (Approval Nos. Gq02268 and 7/2568, respectively), and formal permission to conduct the study was obtained from all four participating hospitals. Prior to data collection, all participants received an information sheet describing the study, after which written informed consent was obtained. No personally identifiable information was collected.

The study population comprised registered nurses working in all clinical wards across four participating hospitals representing both urban and rural healthcare settings. The total target population consisted of 1160 registered nurses, including 498 from Hospital A, 187 from Hospital B, 245 from Hospital C, and 230 from Hospital D. Hospitals were purposively selected based on their geographic location and hospital level to enable comparisons between urban and rural settings. Two urban hospitals (Hospitals A and B) were selected first, followed by two rural hospitals (Hospitals C and D), which represented a regional hospital and a general hospital, respectively. The sample size was initially calculated using the single-population proportion formula based on an estimated WPV prevalence of 47% [21], with a 95% confidence level and a 5% margin of error, yielding a minimum required sample of 383 participants. After accounting for a 10% non-response rate, the target sample size was increased to 420 participants. Because multivariable Poisson regression was used to examine factors associated with WPV frequency, the sample size was considered adequate relative to the number of covariates (approximately 6–7) and deemed sufficient for stable estimation. Eligible participants included registered nurses working full-time for at least 1 year in various roles, such as staff nurses, head nurses, and nurse supervisors. Nurses aged ≥ 21 years who provided informed consent were included, whereas those with incomplete questionnaire data or who withdrew prior to study completion were excluded. During data collection, five nurses from Hospital A withdrew from the study. Consequently, the final sample comprised 415 registered nurses, including 205 from urban hospitals and 210 from rural hospitals. Although the final sample size (*n* = 415) was slightly lower than the target, it remained close to the required sample size and provided balanced representation across the two healthcare settings.

### 2.2. Measurements

Data were collected using a self-administered questionnaire developed based on the standardized Workplace Violence Questionnaire jointly developed by the International Labor Association, International Council of Nurses, World Health Organization, and Public Services International (ILO/ICN/WHO/PSI, 2003), which was subsequently updated by WHO [22], and informed by relevant literature [1,3,17,23,24]. The questionnaire was designed to assess the problems, causes, impacts, and preventive strategies related to WPV against nurses in Thai healthcare settings. The questionnaire consisted of three sections: (i) 13 items assessing demographic and work-related characteristics; (ii) 39 items measuring exposure to WPV, including physical violence (14 items), verbal abuse (11 items), and sexual harassment (14 items); and (iii) 3 items evaluating perceptions of WPV and institutional responses. To ensure content validity, the instrument was reviewed by five nursing experts possessing both academic expertise and active frontline clinical practice. Their practical insights derived from direct experience in patient care helped verify the relevance and applicability of the questionnaire items. Content validity indices were calculated, yielding a content validity index (CVI) of 0.95, indicating excellent content validity. All items retained in the final version met acceptable validity standards. The revised instrument was pilot-tested with 40 nurses outside the main study sample. Following necessary refinements, the final questionnaire was administered to 415 nurses. Internal consistency reliability was assessed using Cronbach’s alpha. The psychological and verbal violence subscales demonstrated excellent reliability (α = 0.955 and α = 0.954, respectively). Although the sexual violence subscale also yielded a high Cronbach’s alpha coefficient, this finding should be interpreted with caution because only two cases of sexual violence were reported. The very low frequency of reported cases limits the statistical power to draw meaningful conclusions regarding sexual violence, particularly in the multivariable analyses. Participants completed the questionnaire during scheduled work hours, with an average completion time of approximately 10 min. To protect participants’ privacy and minimize social desirability bias, questionnaires were completed individually in a private area whenever possible. Participants were instructed not to discuss their responses with supervisors or colleagues during completion, and supervisors were not present while the questionnaires were being completed. Completed questionnaires were returned in sealed envelopes and deposited into a designated collection box to ensure anonymity and confidentiality.

### 2.3. Statistical Analysis

All statistical analyses were performed using Stata version 17.0 (Stata Corp, College Station, TX, USA). Data on categorical variables were expressed as frequencies and percentages, while those on continuous variables were expressed as mean ± standard deviation. WPV was defined as any self-reported experience occurring within the previous 12 months. The 12-month prevalence was calculated as the proportion of participants who reported experiencing WPV during this period (*n* = 415). Patient subgroups were compared using appropriate statistical tests. For categorical variables, the chi-square test was applied, while Fisher’s exact test was used when more than 20% of the expected cell counts were <5. Differences in continuous variables were assessed using the independent samples *t*-test for normally distributed data. All statistical tests were two-tailed, and a *p*-value < 0.05 was considered statistically significant.

### 2.4. WPV Characteristics and Reporting

Analyses and reporting of WPV characteristics were restricted to participants who reported at least one experience of WPV within the past 12 months. Descriptive statistics were used to summarize incident characteristics, with frequencies and percentages reported for each category. For variables with multiple possible responses (e.g., perpetrators, causes, actions taken, organizational responses, and reasons for not reporting), proportions were calculated against the total number of exposed participants; therefore, percentages may exceed 100%. In contrast, for single-response variables (e.g., injury occurrence, treatment duration, timing of incident, and presence of colleagues), proportions were calculated using the total number of participants within each subgroup, and percentages sum to approximately 100%. Variables with sparse data, which comprise categories with very few observations, were summarized descriptively to avoid unstable estimates and unreliable statistical inference; formal statistical comparisons were not performed for these variables.

### 2.5. Multivariable Analysis

Multivariable Poisson regression models were fitted to examine factors associated with WPV frequency. The outcome variable was defined as the total number of WPV events reported over the past 12 months, allowing for multiple incidents per individual. WPV events may involve multiple perpetrators, causes, and contexts within a single episode, and individuals may experience repeated incidents over time. Therefore, the count outcome reflects the overall burden of exposure rather than discrete independent events. Before model fitting, the distribution of the outcome was assessed to confirm its suitability for count data analysis. The outcome had a low-count distribution, with a mean of 0.53 and a variance of 0.49, suggesting equidispersion. The distribution was also assessed for skewness and excess zeros. Multicollinearity among independent variables was evaluated using variance inflation factors, and no evidence of substantial multicollinearity was observed. Robust standard errors were applied to account for potential minor deviations from model assumptions. Covariates were selected based on theoretical considerations and existing literature. To control for potential confounding, the multivariable models were adjusted for age, marital status, work department, work hours, type of patients, level of concern about WPV, and healthcare setting (urban vs. rural). Results were reported as adjusted incidence rate ratios (IRRs) with 95% confidence intervals (CIs). Model fit was evaluated using the Pearson goodness-of-fit test, which indicated adequate fit and no evidence of overdispersion (χ^2^/df ≈ 0.91, *p* = 0.91). Negative binomial regression was performed as a sensitivity analysis and yielded consistent results. The dispersion parameter was close to zero, and the likelihood ratio test was not significant, confirming the appropriateness of the Poisson model.

### 2.6. Psychological Responses and Suggested Preventive Measures

Psychological responses following WPV were analyzed. Descriptive statistics were used to summarize the distribution of psychological responses by type of WPV experienced (physical violence, verbal abuse, and overall exposure), with frequencies and percentages reported for each category. For variables with multiple responses (e.g., psychological responses), proportions were calculated using the number of exposed participants in each subgroup as the denominator. Suggested preventive measures were analyzed using descriptive statistics based on the total study population (*n* = 415). Because participants could select multiple options for these variables, proportions were calculated using the total number of respondents as the denominator. Comparisons between urban and rural settings were performed using the chi-square test or Fisher’s exact test, as appropriate.

## 3. Results

### 3.1. Demographic and Work-Related Characteristics of Participants

In total, 415 participants were included in this study, comprising 205 nurses from urban hospitals and 210 from rural hospitals. Overall, the two groups were broadly comparable in terms of age, mean age, sex, marital status, professional nursing experience, work department, shift schedule, work hours, type of patients/clients served, concern about WPV, WPV reporting procedures, and WPV reduction policies. However, the two groups differed significantly in marital status, work department, type of patients served, and level of concern about WPV. Nurses in rural settings were more likely to be married, whereas a higher proportion of nurses in urban settings were single (*p* = 0.014). Departmental distribution also differed significantly between the two groups (*p* < 0.001), reflecting variation in service organization across settings. Regarding the type of patients served (*p* < 0.001), rural nurses reported caring for more mixed-patient groups. Furthermore, concern about WPV was significantly greater among nurses in urban settings than in rural settings, both in the categorical distribution and in mean scores (mean 3.16 vs. 2.54, *p* < 0.001). These differences indicate variation in both work context and perceived risk of WPV between urban and rural healthcare environments. Table 1 presents details on the demographic and work-related characteristics of participants in urban and rural healthcare settings.

### 3.2. Twelve-Month WPV Prevalence

The 12-month prevalence of WPV among participants is presented in Table 2. Overall, 43.61% of the participants reported experiencing at least one form of WPV at least once, with a significantly higher incidence in urban settings than in rural settings (53.66% vs. 33.81%, *p* < 0.001). Regarding specific types of WPV, physical violence was reported by 13.25% of participants, with a significantly higher prevalence in urban settings than in rural settings (16.59% vs. 10.00%, *p* = 0.048). Verbal abuse was the most commonly reported type of WPV, accounting for 41.20% of cases, with a significantly higher prevalence in urban than in rural settings (51.71% vs. 30.95%, *p* < 0.001). Sexual harassment was rare, reported by only 0.98% of urban nurses and none of rural nurses (*p* = 0.24).

### 3.3. Characteristics and Reporting of WPV Incidents

Total percentages are based on the total number of participants (*n* = 181). Subcategory percentages were calculated within the relevant violence type (physical, *n* = 55; verbal, *n* = 171). For variables with multiple possible responses, percentages may exceed 100%. Among participants who reported any WPV (*n* = 181), 55 (30.39%) experienced physical violence (Table 3). No significant differences were observed between urban and rural settings in weapon use, injury occurrence, treatment duration, perpetrator, time of incident, presence of colleagues during the incident, actions taken after the incident, workplace investigation, organizational response, and reasons for not reporting. The most frequently reported cause of physical violence was illness or medical condition (67.27%), followed by dissatisfaction/anger (50.91%) and alcohol or substance use (41.82%). When comparing causes of physical violence between settings, alcohol or substance use was the only factor that differed significantly between urban and rural settings, with a significantly higher prevalence in urban settings (58.82% vs. 14.29%, *p* = 0.001) (Table 3).

Among participants who reported experiencing any WPV (*n* = 181), 171 (94.48%) experienced verbal abuse. The most frequently reported perpetrator was patients’/clients’ relatives (61.99%), followed by patients/clients (59.06%) and colleagues (18.71%). A significantly higher proportion of patients/clients as perpetrators was observed in urban settings (66.04% vs. 47.69%, *p* = 0.025). In contrast, colleagues as perpetrators were reported more frequently in rural settings (27.69% vs. 13.21%, *p* = 0.026). Dissatisfaction/anger was the most frequently reported cause of verbal abuse (84.21%), followed by illness or medical condition (45.03%) and anxiety (40.35%). Dissatisfaction/anger (88.68% vs. 76.92%, *p* = 0.041) and illness or medical condition as causes of verbal abuse were reported more frequently in urban settings (50.94% vs. 35.38%, *p* = 0.047). For actions taken after the incident, the most commonly reported response was discussing the incident with coworkers (75.44%), followed by avoiding/leaving the situation (43.27%) and verbally reporting the incident to a supervisor (33.92%). Nurses in urban settings were significantly more likely to avoid or leave the situation than those in rural settings (50.00% vs. 32.31%, *p* = 0.023). The most frequently reported reason for not reporting an incident was perceived uselessness (31.58%), followed by complex procedures (18.13%) and lack of time (14.04%). Complex procedures were reported significantly more frequently in urban settings than in rural settings (24.53% vs. 7.69%, *p* = 0.006). No significant differences were observed between urban and rural settings in time, the presence of colleagues during the incident, workplace investigation, or organizational response (Table 3).

Furthermore, two participants from urban settings (1.10%) reported experiencing sexual harassment. The separate incidents occurred in inpatient wards during the evening shift (6:00 p.m.–11:59 p.m.). In both cases, the perpetrators were patients/clients, and the reported cause was illness or medical condition. The incidents occurred in the presence of colleagues. Following their respective incidents, both participants discussed them with coworkers, with one participant verbally reporting the incident to a supervisor. One participant avoided or left the situation and discussed the incident with family or friends. The other participant did not report the incident due to complex procedures and because they perceived reporting as useless.

### 3.4. Factors Associated with WPV

Table 4 presents factors associated with WPV among nurses. After adjustment for potential confounders, age, marital status, department of work, work hours, type of patients served, and level of concern remained significantly associated with WPV. Compared with nurses aged ≥ 51 years, those aged 21–30 years had significantly higher incidence rates of WPV (Adjusted [Adj] IRR = 3.31, 95% CI: 1.28–8.56). Similarly, nurses aged 31–40 years (Adj IRR = 3.08, 95% CI: 1.12–8.47) and 41–50 years (Adj IRR = 4.76, 95% CI: 1.52–14.87) also showed significantly higher incidence rates of WPV compared with the ≥51 years age group. When stratified by healthcare setting, nurses aged 21–30 years in urban settings had a significantly higher incidence rate of WPV than those aged ≥ 51 years (Adj IRR = 3.07, 95% CI: 1.04–9.07). However, no significant association was observed between age 21–30 years and WPV incidence among nurses in rural settings.

Divorced or separated nurses had a significantly higher incidence of WPV than married nurses (Adj IRR = 3.05, 95% CI: 1.54–6.04). When stratified by healthcare setting, divorced or separated nurses in urban settings showed a significantly higher incidence rate of WPV (Adj IRR = 3.78, 95% CI: 1.66–8.61), whereas no significant association was observed between divorced or separated status and WPV incidence in rural settings.

Nurses working in the emergency department (Adj IRR = 2.47, 95% CI: 1.11–5.47) and the medical ward (Adj IRR = 3.33, 95% CI: 1.60–6.91) had significantly higher incidence rates of WPV than those working in private/VIP wards. When stratified by healthcare setting, nurses working in medical wards in rural settings had a significantly higher incidence rate of WPV than those in private/VIP wards (IRR = 3.24, 95% CI: 1.20–8.71).

Nurses working both day and night shifts had significantly higher incidence rates of workplace violence than those working only day shifts (Adj IRR = 2.03, 95% CI: 1.13–3.64). This association remained significant in urban settings (Adj IRR = 2.51, 95% CI: 1.22–5.15) but was not significant in rural settings.

Nurses caring for male-only patient groups had a significantly higher incidence rate of WPV than those caring for mixed-patient groups (Adj IRR = 1.87, 95% CI: 1.28–2.74).

Nurses who reported greater concern about WPV had significantly higher incidence rates than those who were not concerned. Nurses who were slightly concerned (Adj IRR = 2.17, 95% CI: 1.12–4.20), moderately concerned (Adj IRR = 2.51, 95% CI: 1.36–4.63), very concerned (Adj IRR = 4.11, 95% CI: 2.21–7.63), and extremely concerned (Adj IRR = 4.57, 95% CI: 2.34–8.93) had significantly higher incidence rates of WPV than those who were not concerned. Finally, nurses in urban settings had a significantly higher risk of experiencing WPV than those in rural settings (Adj IRR = 1.68, 95% CI: 1.28–2.20). Thus, urban healthcare settings are associated with a 1.68 times greater risk of WPV after adjusting for other factors (Table 4).

### 3.5. Psychological Responses Following WPV

Among nurses who experienced physical violence (*n* = 55), the most commonly reported psychological response was hypervigilance or heightened alertness (76.36%), followed by avoidance of discussing the incident (52.73%), reluctance to come to work (38.18%), and loss of professional confidence (38.18%). Nurses in urban settings were significantly more likely to report recurrent thoughts about the incident (47.06% vs. 19.05%, *p* = 0.036) and an intention to resign or transfer (50.00% vs. 14.29%, *p* = 0.007) than those in rural settings. No significant differences were observed for other psychological responses. The psychological responses of nurses who experienced verbal abuse (*n* = 171) in order of frequency were hypervigilance or heightened alertness (59.65%), avoidance of discussing the incident (56.14%), recurrent thoughts about the incident (47.37%), and intention to resign or transfer (43.27%). Nurses in urban settings were significantly more likely to report an intention to resign or transfer (50.94% vs. 30.77%, *p* = 0.010) and a loss of professional confidence (48.11% vs. 29.23%, *p* = 0.015) than those in rural settings. No significant differences were observed between settings for other psychological responses (Table 5).

### 3.6. Nurses’ Recommendations for Preventing WPV in Healthcare Settings

Nurses’ recommendations for preventing WPV in healthcare settings are summarized in Table 6. Overall, the most frequently recommended measure was clear disciplinary actions (64.82%), followed by strengthening hospital security systems (50.84%) and increasing staffing levels (50.12%). Urban-setting nurses were significantly more likely than rural-based ones to recommend increasing staffing levels (58.54% vs. 41.90%, *p* = 0.001), improving the workplace environment (54.15% vs. 37.14%, *p* = 0.001), strengthening security systems (56.10% vs. 45.71%, *p* = 0.034), and support from organizational leadership (43.90% vs. 26.67%, *p* < 0.001). These findings further reflect the context-specific needs in high-density, high-demand urban hospitals.

## 4. Discussion

### 4.1. Differences in Demographic and Work-Related Characteristics Between Urban and Rural Healthcare Settings

This study provides a comparative analysis of WPV incidence between nurses in urban and rural healthcare settings in Thailand, highlighting prevalence, contributing factors, psychological impacts, and preventive measures. Although direct comparative evidence between urban and rural contexts is limited, and geographic interpretations should be made cautiously [25], our findings reveal meaningful differences that are highly relevant to understanding WPV risk. Although age distribution did not differ significantly between urban and rural nurses, marital status varied significantly, with a higher proportion of rural nurses being married. Notably, work-related characteristics demonstrated substantial variation across settings. Urban hospitals had a higher proportion of nurses working in medical wards, indicating frequent interaction with patients with chronic and complex conditions, particularly among older populations. These wards are associated with higher care demands, longer waiting times, and increased stress for both patients and families, all of which are recognized contributors to WPV, consistent with evidence from emergency wards internationally and within Thailand [1,11]. In contrast, rural-based nurses more frequently managed mixed-patient care models, covering a broader range of conditions within the same unit. This finding may reflect differences in service organization between rural and urban hospitals. However, evidence explaining how these organizational differences influence workplace violence remains limited [10,26].

### 4.2. Prevalence and Types of WPV

In this study, 43.61% of 415 nurses reported experiencing at least one incident of WPV within the previous 12 months. Verbal abuse was the most frequently reported form (41.20%), followed by physical violence (13.25%), while sexual harassment was rare. The low reported incidence of sexual harassment and other forms of violence may reflect the perception among Thai nurses that reporting is often ineffective or unlikely to result in meaningful action, which was the most commonly cited reason for non-reporting. Compared with international evidence, the prevalence observed in this study is lower. An umbrella review found an overall WPV prevalence of 58.7%, with verbal abuse at 66.8% and physical violence at 20.8% [27]. Similarly, another study reported higher rates of physical violence (36.4%) and non-physical violence (66.9%) among healthcare workers globally [28]. Within Thailand, the slightly higher prevalence of physical WPV in this study compared with previous nationwide studies (9.1%) may be attributed to the inclusion of multiple large urban hospitals serving diverse patient populations. In contrast, prior studies were conducted in single university hospitals with controlled patient numbers [10]. Interestingly, the overall prevalence of physical violence in the present study (13.25%) was lower than the pooled 1-year prevalence estimate reported in a systematic review (19.33%; 95% CI, 16.49–22.53%) [29]. However, among urban hospitals specifically, the prevalence (16.59%) was comparable to the pooled estimate, demonstrating the influence of local context on WPV exposure. High heterogeneity across studies suggests that differences in setting, measurement methods, cultural factors, and reporting behaviors may explain the variability in prevalence rates [29].

### 4.3. Characteristics and Reporting of WPV Incidents

Urban–rural differences were notable among the 181 nurses who experienced WPV in the preceding 12 months. Verbal abuse (58.56% vs. 35.91%) and physical violence (18.78% vs. 11.60%) were more frequent in urban hospitals, reflecting a higher risk in dense patient settings. Temporal patterns indicate that physical violence in urban hospitals peaked in the evening, coinciding with visiting hours and interactions with patients’ relatives, whereas in rural hospitals, incidents were more common between 12:00 a.m. and 6:59 a.m., often linked to patient confusion or disease-related behaviors that patients could not recall [10]. While illness or medical conditions remain the predominant causes of physical violence in both urban and rural hospitals, urban incidents were more frequently associated with patients and their relatives (17.65% vs. 0.00%), alcohol or substance use (58.82% vs. 14.29%), and weapon use (23.53% vs. 9.52%). Evidence from both Thai and international reports indicate that WPV initiated by patients and their relatives is strongly associated with intense emotional responses and high expectations [30,31]. Alcohol or substance use was another notable contributor to WPV in urban hospitals, consistent with prior systematic reviews and studies in Thai metropolitan emergency departments [11,32]. Although injury occurrence and treatment duration did not differ significantly between settings, physical violence perpetrated by patients or their relatives, alcohol or substance use, and weapon use may impose greater negative psychological and occupational impacts on nurses in urban hospitals than those in rural hospitals, where patient-related factors such as disease-related confusion predominate.

Verbal abuse mirrored the urban–rural differences in the distribution of physical violence but was more widespread. Patients and their relatives were the primary perpetrators in both urban and rural settings, although the pattern was more pronounced in urban hospitals (66.04% vs. 55.38%). Notably, colleague-perpetrated verbal abuse was higher in rural settings (27.69% vs. 13.21%, *p* = 0.026), possibly reflecting a local culture influenced by lighter workloads and different interpersonal dynamics. Although evidence specifically explaining this rural–urban difference remains limited, workplace bullying among nurses has been reported to be associated with multiple factors, including demographics, personality, organizational culture, work characteristics, and leadership and hierarchical structures [33]. The main causes of verbal abuse were dissatisfaction or anger, although alcohol or substance use contributed slightly. The findings of the present study align with existing Thai and international evidence regarding the primary perpetrators and contributing factors of WPV among nurses [9,31].

Following incidents of WPV, both urban and rural nurses most frequently discussed the events with coworkers (58.82–76.92% across all types of WPV), highlighting the importance of peer support and shared understanding. Urban nurses, however, were more likely to avoid or leave violent situations than rural nurses (50.00% vs. 32.31%, *p* = 0.023) and were more likely to cite complex reporting procedures as reasons for non-reporting (24.53% vs. 7.69%, *p* = 0.006). Notably, nurses across both settings cited the perceived uselessness of reporting as the primary barrier to reporting, reflecting common concerns about the effectiveness and complexity of reporting systems. This underscores the critical need for streamlined and effective reporting protocols to provide timely support and protection for nurses experiencing WPV. These findings align with international evidence indicating that minor assaults are frequently underreported and that healthcare workers may accept some level of abuse as part of the job [19,24,25,34,35]. A recent scoping review (2026) reported that underreporting exceeds 50% in many studies, with the most frequent hindrance being the belief that reporting is useless. This aligns closely with the findings of the present study, most frequently in Thai emergency and critical care units, where only 12.3% of incidents were officially reported, with the main reason for not reporting being the perception that it would not lead to change [27]. In addition, complex reporting procedures were identified as another major barrier to reporting in both urban and rural hospitals, accounting for 44.12% and 38.10% of responses, respectively. This finding is consistent with a recent systematic review, which identified organizational barriers, including the lack of clear policies and procedures, inadequate training, and inefficient or non-user-friendly reporting systems, as important contributors to the underreporting of workplace violence among nurses. The review also highlighted that management-related factors, such as the lack of visible improvements following reports, a non-supportive reporting culture, and insufficient accountability for perpetrators, further discouraged nurses from reporting WPV incidents. These findings suggest that simplifying reporting procedures, strengthening organizational support, and establishing responsive reporting systems may improve reporting behaviors among nurses [19]. Overall, WPV among Thai nurses is highly prevalent and context-dependent, with urban hospitals carrying greater exposure and complexity. These findings highlight the need for tailored preventive measures. Furthermore, future research in Thailand must prioritize elucidating the underlying reasons why nurses often choose not to report incidents despite available reporting channels [34].

### 4.4. Factors Associated with WPV

In the present study, urban location was a significantly associated with WPV among nurses (Adj IRR = 1.68), likely due to the concentration of healthcare resources in urban hospitals, including tertiary centers, specialized units, and private clinics. Such scenarios result in complex patient flows and high volumes of chronic, acute, and emergency cases. In contrast, rural hospitals administer lower-volume, general care using mixed-patient models [36]. These structural differences likely contribute to the urban–rural disparity in WPV risk, consistent with prior evidence linking overcrowding, complex caseloads, understaffing, and patient turnover in urban settings to increased WPV incidence [1,10,27,28,37].

However, demographic factors influenced WPV inconsistently. Younger nurses (aged 21–30 years) in urban hospitals were at higher risk than those aged ≥ 51 (Adj IRR = 3.07), while divorced or separated nurses exhibited elevated incidence ratios, reflecting prior evidence of vulnerability among younger or less experienced staff [10,14,24]. However, age and marital status were not consistently associated with WPV across different contexts, suggesting that structural and work-related factors may play a more important role in WPV exposure [38]. Work-related characteristics were strongly associated with WPV among nurses. Those working in medical wards (adjusted IRR = 3.33) and emergency departments (adjusted IRR = 2.47) had significantly higher rates of WPV than nurses in other departments across both urban and rural healthcare settings. High-density wards managing large patient volumes, complex cases, and crowded conditions [10,34] were more exposed than private/VIP wards, which reflect patient loads and care intensity akin to those in high-standard private hospitals. Medical wards in Thailand manage patients with various chronic and infectious diseases, as well as suicide attempts and substance-related conditions, resulting in a high patient density and complex interactions with families and staff, thereby elevating stress and the likelihood of aggression. Similarly, international evidence identifies ERs, trauma, psychiatric, and outpatient units with high patient turnover as WPV hotspots [24,34,37]. Shift work and combined day–night schedules also increased exposure, underscoring the impact of work patterns [1,8,10,11]. A high level of concern about WPV was strongly associated with WPV exposure, with nurses reporting extreme concern experiencing 4.87 times higher incidence rates of WPV than those reporting low concern. Interestingly, sex was not significantly associated with WPV in this study, in contrast to previous studies reporting a higher occurrence of WPV among male nurses [9]. This discrepancy may reflect the comprehensive nature of the current study, which included nearly the entire nursing population in the selected hospitals, providing a more accurate representation of real-world exposure. Furthermore, the findings of the current study support the implementation of context-specific preventive and management strategies for WPV, informed by empirical evidence. Interventions should account for service-system differences and demographic risk factors, prioritizing actions by urgency and impact on nurse well-being, and focusing on high-risk wards, including targeted staff training and organizational strategies for the departments most exposed to violence.

### 4.5. Psychological Impacts of WPV Among Thai Nurses

The psychological impacts of WPV among Thai nurses were substantial. Hypervigilance was the most frequently reported response overall (66.30%), especially immediately following physical violence (76.36%) compared with verbal abuse (59.65%). Over 40% of affected nurses reported intentions to resign or transfer (41.99%), reluctance to come to work (41.99%), and loss of professional confidence (41.44%). When stratified by violence type, intention to resign or transfer and loss of professional confidence were higher among nurses exposed to physical violence (50.00% and 44.12%, respectively) and verbal abuse (50.94% and 48.11%, respectively), with urban nurses consistently reporting greater severity. These findings corroborate international evidence that WPV imposes significant emotional and occupational toll on nurses, including increased burnout, post-traumatic stress disorder, and reduced professional engagement. Consistent with prior studies, WPV is linked to psychological distress manifestations, such as hypervigilance, avoidance, diminished confidence, and higher turnover intentions [27,28,39,40]. This underscores the urgent need for organizational interventions to mitigate the significant psychological and occupational impacts of WPV on nurses. Senior management and all relevant stakeholders must implement targeted preventive measures that actively address the identified problems and their urgency.

### 4.6. Nurses’ Recommendations for Preventing WPV

According to the 415 nurses surveyed, clear, enforceable disciplinary actions were the most desired preventive measure in both urban and rural settings. For urban nurses, the second and third most desirable measures were increasing staffing levels (58.54% vs. 41.90%, *p* = 0.001) and improving the workplace environment (54.15% vs. 37.14%, *p* = 0.001), indicating the associated effects of acute workforce shortages and operational pressures. Additional urban priorities included strengthening hospital security systems (56.10% vs. 45.71%, *p* = 0.034) and organizational leadership support (43.90% vs. 26.67%, *p* < 0.001). In contrast, rural nurses emphasized further training on violence management (45.71% vs. 42.44%, *p* = 0.502) and developing formal violence prevention plans (41.90% vs. 47.32%, *p* = 0.267), indicating a greater demand for capacity building and procedural preparedness in smaller, mixed-patient settings with lower patient density. Collectively, these findings highlight the urgent need for context-specific preventive strategies to address WPV against nurses in Thailand. While urban hospitals require structural and leadership-focused interventions to enhance staffing, organizational support, environmental safety, and security, rural hospitals prioritize staff training and formalized prevention planning. Both contexts can implement preventive measures simultaneously if administrators and stakeholders recognize the urgency, listen to frontline nurses’ experiences, and act decisively. This evidence-based approach is critical to preventing deterioration in nurse well-being and preserving the quality of patient care, including disease prevention, treatment, and rehabilitation [27,28,41].

## 5. Strengths and Limitations

This study comprehensively examined WPV among nurses in both urban and rural Thai hospitals, identifying problems, causes, psychological and occupational impacts, and preventive strategies. Data were collected directly from 415 practicing nurses across multiple wards, ensuring relevance and immediacy of responses. The questionnaire was developed after conducting a thorough literature review and subsequently validated by five experts with ongoing clinical experience in urban and rural settings, ensuring an accurate reflection of real-world challenges. Paper-based data collection facilitated on-site verification and follow-up, enhancing data completeness while protecting participant confidentiality. By incorporating nurses’ perspectives, including their perceptions of reporting futility, the study provides nuanced insights that go beyond administrative or managerial records. The comparative analysis between urban and rural hospitals highlights context-specific differences, supporting a systematic understanding of WPV across diverse healthcare settings. The collaboration between hospital management and staff facilitated access and participation, further strengthening the quality and reliability of the data.

However, the study sample included only four hospitals (two urban and two rural), limiting the generalizability of the findings to healthcare facilities nationwide. Findings from urban hospitals reflect high-volume settings but may not represent all urban contexts, while outcomes in rural settings may not adequately capture the diversity of smaller or remote facilities. Certain individual-level factors, such as alcohol or substance use among nurses, were underassessed, and cyber- or technology-mediated violence was not sufficiently addressed. Retrospective self-reporting may introduce recall bias, and some associations, such as concern about WPV, may partly reflect previous experiences with WPV rather than factors that preceded WPV exposure. Although they facilitate on-site verification, paper-based questionnaires may create confidentiality concerns. However, clear instructions and direct submission options mitigated this issue. Furthermore, non-participation in some wards prevented comprehensive coverage. Future investigations, particularly the ongoing phase 2 qualitative study, should conduct a deeper exploration of the experiences of affected nurses and administrators to obtain complementary insights and refine context-specific preventive strategies for WPV.

## 6. Conclusions

WPV is highly prevalent and context-dependent among Thai nurses, with urban-based nurses experiencing higher exposure and more severe psychological impacts, including hypervigilance, avoidance, loss of professional confidence, and intentions to resign or transfer. Physical violence in urban hospitals increases with patient density, complex cases, and interactions with patients and their relatives, including alcohol- or substance-related incidents. In contrast, rural-based nurses’ experiences of WPV were primarily linked to patient illness or confusion. Nurses across both settings emphasized the implementation of clear, enforceable disciplinary actions as the most critical preventive measure, reflecting widespread perceptions that reporting is often useless due to cumbersome procedures and weak enforcement. Urban-setting nurses prioritized structural and leadership interventions—such as enhanced staffing, improved environments, stronger security, and organizational support—while rural-based nurses identified staff training and formal prevention planning as interventions, demonstrating the need for context-specific strategies. These findings underscore the urgent responsibility of nursing leadership and all stakeholders to actively address the problem, implement effective measures, and streamline reporting systems. Addressing WPV decisively protects nurses’ well-being, fosters workforce resilience, and improves quality patient care.

## Figures and Tables

**Table 1:** Demographic and work-related characteristics of participants in urban and rural healthcare settings (*n* = 415).

Characteristic	Urban, *n* (%)			Rural, *n* (%)			*p*-Value
	Total (*n* = 205)	Hospital A (*n* = 100)	Hospital B (*n* = 105)	Total (*n* = 210)	Hospital C (*n* = 105)	Hospital D (*n* = 105)	
Age (years)							0.594 ^†^
21–30	113 (55.12)	55 (55.00)	58 (55.24)	119 (56.67)	73 (69.53)	46 (43.81)	
31–40	54 (26.34)	33 (33.00)	21 (20.00)	62 (29.52)	26 (24.76)	36 (34.28)	
41–50	24 (11.71)	5 (5.00)	19 (18.09)	19 (9.05)	4 (3.81)	15 (14.29)	
≥51	14 (6.83)	7 (7.00)	7 (6.67)	10 (4.76)	2 (1.90)	8 (7.62)	
Mean ± SD	31.28 ± 8.62	31.28 ± 8.62	33.86 ± 9.76	31.56 ± 8.59	29.03 ± 6.69	34.12 ± 9.52	0.237 ^‡^
Sex							0.395 ^†^
Female	195 (95.12)	95 (95.00)	100 (95.24)	205 (97.62)	102 (97.14)	103 (98.10)	
Male	8 (3.90)	4 (4.00)	4 (3.81)	4 (1.90)	3 (2.86)	1 (0.95)	
Sexual minority	2 (0.98)	1 (1.00)	1 (0.95)	1 (0.48)	0 (0.00)	1 (0.95)	
Marital Status							0.014 ^†^
Single	147 (71.70)	79 (79.00)	68 (64.77)	130 (61.90)	72 (68.57)	58 (55.24)	
Married	50 (24.39)	19 (19.00)	31 (29.52)	77 (36.67)	33 (31.43)	44 (41.90)	
Divorced/Separated	6 (2.93)	1 (1.00)	5 (4.76)	3 (1.43)	0 (00.00)	3 (2.86)	
Widowed	2 (0.98)	1 (1.00)	1 (0.95)	0 (00.00)	0 (00.00)	0 (00.00)	
Professional Nursing Experience							0.392 ^†^
Less than 1 year	22 (10.73)	14 (14.00)	8 (7.62)	29 (13.81)	21 (20.00)	8 (7.62)	
1–5 years	62 (30.25)	30 (30.00)	32 (30.47)	67 (31.91)	34 (32.39)	33 (31.43)	
6–10 years	58 (28.30)	34 (34.00)	24 (22.86)	52 (24.76)	30 (28.57)	22 (20.95)	
11–15 years	21(10.24)	8 (8.00)	13 (12.38)	31 (14.76)	13 (12.38)	18 (17.14)	
16–20 years	7 (3.41)	4 (4.00)	3 (2.86)	4 (1.90)	1 (0.95)	3 (2.86)	
20 years and above	35 (17.07)	10 (10.00)	25 (23.81)	27 (12.86)	6 (5.71)	21 (20.00)	
Work Department							
Emergency department (ER)	23 (11.22)	6 (6.00)	17 (16.20)	20 (9.52)	4 (3.81)	16 (15.24)	<0.001 ^†^
Outpatient department (OPD)	13 (6.34)	6 (6.00)	7 (6.67)	19 (9.05)	3 (2.86)	16 (15.24)	
Intensive care unit (ICU)	34 (16.58)	16 (16.00)	18 (17.14)	35 (16.66)	26 (24.75)	9 (8.57)	
Medical ward	67 (32.68)	32 (32.00)	35 (33.33)	27 (12.86)	19 (18.10)	8 (7.62)	
Surgical ward	33 (16.10)	15 (15.00)	18 (17.14)	29 (13.81)	18 (17.14)	11 (10.48)	
Pediatric ward	15 (7.32)	11 (11.00)	4 (3.81)	27 (12.86)	17 (16.19)	10 (9.52)	
Private/VIP ward	9 (4.39)	5 (5.00)	4 (3.81)	24 (11.43)	7 (6.67)	17 (16.19)	
Other ^¥^	11 (5.37)	9 (9.00)	2 (1.90)	29 (13.81)	11 (10.48)	18 (17.14)	
Shift/Work Schedule							0.327 ^†^
No shift work	19 (9.27)	10 (10.00)	9 (8.57)	14 (6.67)	1 (0.95)	13 (12.38)	
Work in shifts	186 (90.73)	90 (90.00)	96 (91.43)	196 (93.33)	104 (99.05)	92 (87.62)	
Work Hours							0.493 ^†^
Both day and night	177 (86.34)	88 (88.00)	89 (84.76)	186 (88.57)	102 (97.14)	84 (80.00)	
Day only	28 (13.66)	12 (12.00)	16 (15.24)	24 (11.43)	3 (2.86)	21 (20.00)	
Type of Patients/Clients Served							
Male only	45 (21.95)	18 (18.00)	27 (25.71)	9 (4.29)	7 (6.67)	2 (1.90)	<0.001 ^†^
Female only	41 (20.00)	24 (24.00)	17 (16.19)	25 (11.90)	18 (17.14)	7 (6.67)	
Pediatric only	10 (4.88)	6 (6.00)	4 (3.81)	17 (8.10)	6 (5.71)	11 (10.48)	
Postpartum only	3 (1.46)	3 (3.00)	0 (0.00)	9 (4.29)	9 (8.57)	0 (0.00)	
Mixed-patient groups	106 (51.71)	49 (49.00)	57 (54.29)	150 (71.42)	65 (61.91)	85 (80.95)	
Concern About Workplace Violence							
Not concerned	19 (9.27)	12 (12.00)	7 (6.67)	45 (21.43)	24 (22.86)	21 (20.00)	<0.001 ^†^
Slightly concerned	31 (15.12)	15 (15.00)	16 (15.24)	52 (24.76)	26 (24.76)	26 (24.76)	
Moderately concerned	78 (38.05)	45 (45.00)	33 (31.43)	77 (36.67)	32 (30.48)	45 (42.86)	
Very concerned	52 (25.36)	21 (21.00)	31 (29.52)	26 (12.38)	17 (16.19)	9 (8.57)	
Extremely concerned	25 (12.20)	7 (7.00)	18 (17.14)	10 (4.76)	6 (5.71)	4 (3.81)	
Mean ± SD	3.16 ± 1.12	2.96 ± 1.06	3.35 ± 1.13	2.54 ± 1.10	2.57 ± 1.18	2.51 ± 1.02	<0.001 ^‡^
Workplace Violence Reporting Procedure							0.088 ^†^
No	21 (10.24)	9 (9.00)	12 (11.43)	12 (5.71)	4 (3.81)	8 (7.62)	
Yes	184 (89.76)	91 (91.00)	93 (88.57)	198 (94.29)	101 (96.19)	97 (92.38)	
Workplace Violence Reduction Policies							0.357 ^†^
No	22 (10.73)	8 (8.00)	14 (13.33)	17 (8.10)	6 (5.71)	11 (10.48)	
Yes	183 (89.27)	92 (92.00)	91 (86.67)	193 (91.90)	99 (94.29)	94 (89.52)	

^†^ Differences in categorical variables on the chi-square test or Fisher’s exact test; ^‡^ Differences in mean values on the independent *t*-test; ^¥^ Other departments include labor room, postpartum ward, gynecology, ear–eye–nose–throat unit, and operating room, among others, that were grouped together for analysis because of their small sample sizes.

**Table 2:** Twelve-month prevalence of workplace violence among nurses by setting (Overall: *n* = 415; Urban: *n* = 205; Rural: *n* = 210).

Type of Workplace Violence	Total	Urban	Rural	*p*-Value
	*n* (%)	*n* (%)	*n* (%)	
Any workplace violence (Multiple responses)	181 (43.61)	110 (53.66)	71 (33.81)	<0.001 ^†^
Physical violence	55 (13.25)	34 (16.59)	21 (10.00)	0.048 ^†^
Verbal abuse	171 (41.20)	106 (51.71)	65 (30.95)	<0.001 ^†^
Sexual harassment	2 (0.48)	2 (0.98)	0 (0.00)	0.24 ^†^

^†^ Differences in categorical variables on the chi-square test or Fisher’s exact test.

**Table 3:** Characteristics and reporting of workplace violence prevalence among nurses who experienced violence in the past 12 months, by setting (*n* = 181).

Characteristics	Total	Urban	Rural	*p*-Value
	*n* (%)	*n* (%)	*n* (%)	
Physical violence	55 (30.39)	34 (18.78)	21 (11.60)	<0.001
Weapon used	10 (18.18)	8 (23.53)	2 (9.52)	0.191
Injury occurred	20 (36.36)	12(35.29)	8 (38.10)	0.834
Treatment duration				0.664
none	49 (89.09)	31 (91.18)	18 (85.71)	
1–7 days	6 (10.91)	3 (8.82)	3 (14.29)	
Perpetrator of physical violence (Multiple responses)				
Patient/Client	47 (85.45)	29 (85.29)	18 (85.71)	1.000
Patient’s/Client’s relative	6 (10.91)	6 (17.65)	0 (0.00)	0.072
Colleague	1 (1.82)	1 (2.94)	0 (0.00)	1.000
Physician	1 (1.82)	0 (0.00)	1 (4.76)	0.382
Supervisor	1 (1.82)	0 (0.00)	1 (4.76)	0.382
Causes of physical violence (Multiple responses)				
Dissatisfaction/anger	28 (50.91)	19 (55.88)	9 (42.86)	0.348
Anxiety	10 (18.18)	7 (20.59)	3 (14.29)	0.725
Alcohol or substance use	23 (41.82)	20 (58.82)	3 (14.29)	0.001
Illness or medical condition	37 (67.27)	23 (67.65)	14 (66.67)	0.940
Time pressure or urgency	4 (7.27)	3 (8.82)	1 (4.76)	1.000
Communication problems	3 (5.45)	2 (5.88)	1 (4.76)	1.000
Time of incident (Multiple responses)				0.595
7:00 a.m.–12:59 p.m.	4 (7.27)	3 (8.82)	1(4.76)	
1:00 p.m.–5:59 p.m.	12 (21.82)	7 (20.59)	5 (23.81)	
6:00 p.m.–11:59 p.m.	11 (20.00)	9 (26.47)	2 (9.52)	
12:00 a.m.–6.59 a.m.	12 (21.82)	6 (17.65)	6 (28.57)	
Cannot recall	15 (27.27)	8 (23.53)	7 (33.33)	
Multiple time periods	1 (1.82)	1 (2.94)	0 (00.00)	
Presence of colleagues during the incident				1.000
No	8 (14.55)	5 (14.71)	3 (14.29)	
Yes	47 (85.45)	29 (85.29)	18 (85.71)	
Actions taken after the incident (Multiple responses)				
No action taken	9 (16.36)	5 (14.71)	4 (19.05)	0.719
Avoided/left the situation	14 (25.45)	11 (32.35)	3 (14.29)	0.135
Discussed with coworkers	35 (63.64)	20 (58.82)	15 (71.43)	0.345
Discussed with family/friends	6 (10.91)	0 (00.00)	6 (17.65)	0.072
Verbal report to supervisor	23 (41.82)	17 (50.00)	6 (28.57)	0.118
Written report to supervisor	7 (12.73)	5 (14.71)	2 (9.52)	0.696
Police/legal action	3 (5.45)	3 (8.82)	0 (00.00)	0.279
Workplace investigation				0.744
No investigation	28 (50.91)	16 (47.06)	12 (57.14)	
Investigation conducted	26 (47.27)	17 (50.00)	9 (42.86)	
Not required	1 (1.82)	1 (2.94)	0 (00.00)	
Organizational response (Multiple responses)				
No action	6 (10.91)	4 (11.76)	2 (9.52)	1.000
Verbal warning	20 (36.36)	13 (38.24)	7 (33.33)	0.714
Written documentation	11 (20.00)	7 (20.59)	4 (19.05)	1.000
Disciplinary action	2 (3.64)	2 (5.88)	0 (00.00)	0.519
Legal action	1 (1.82)	1 (2.94)	0 (00.00)	1.000
Not required	4 (7.27)	3 (8.82)	1 (4.76)	1.000
Reasons for not reporting (Multiple responses)				
Complex procedures	23 (41.82)	15 (44.12)	8 (38.10)	0.660
Lack of time	12 (21.82)	7 (20.59)	5 (23.81)	1.000
Lack of reporting knowledge	7 (12.73)	6 (17.65)	1 (4.76)	0.232
Fear of consequences	4 (7.27)	2 (5.88)	2 (9.52)	0.632
Perceived as useless	22 (40.00)	17 (50.00)	5 (23.81)	0.054
Verbal abuse	171 (94.48)	106 (58.56)	65 (35.91)	<0.001
Perpetrator of verbal abuse (Multiple responses)				
Patient/Client	101 (59.06)	70 (66.04)	31 (47.69)	0.025
Patient’s/Client’s relative	106 (61.99)	70 (66.04)	36 (55.38)	0.164
Colleague	32 (18.71)	14 (13.21)	18 (27.69)	0.026
Physician	16 (9.36)	8 (7.55)	8 (12.31)	0.299
Supervisor	11 (6.43)	7 (6.60)	4 (6.15)	0.907
Causes of verbal abuse (Multiple responses)				
Dissatisfaction/anger	144 (84.21)	94 (88.68)	50 (76.92)	0.041
Anxiety	69 (40.35)	43 (40.57)	26 (40.00)	0.942
Alcohol or substance use	45 (26.32)	32 (30.19)	13 (20.00)	0.142
Illness or medical condition	37 (45.03)	54 (50.94)	23 (35.38)	0.047
Time pressure or urgency	45 (26.32)	24 (22.64)	21 (32.31)	0.164
Communication problems	64 (37.43)	42 (39.62)	22 (33.85)	0.449
Prior conflict	7 (4.09)	5 (4.72)	2 (3.08)	0.710
Time of incident				0.308
7:00 a.m.–12:59 p.m.	30 (17.54)	19 (17.92)	11 (16.92)	
1:00 p.m.–5:59 p.m.	36 (21.05)	25 (23.58)	11 (16.92)	
6:00 p.m.–11:59 p.m.	22 (12.87)	14 (13.21)	8 (12.31)	
12:00 a.m.–6.59 a.m.	6 (3.51)	2 (1.89)	4 (6.15)	
Cannot recall	64 (37.43)	41 (38.68)	23 (35.38)	
Multiple time periods	13 (7.60)	5 (4.72)	8 (12.31)	
Presence of colleagues during the incident				0.625
No	21 (12.28)	12 (11.32)	9 (13.85)	
Yes	150 (87.72)	94 (88.68)	56 (86.15)	
Actions taken after the incident (Multiple responses)				
No action taken	20 (11.70)	11 (10.38)	9 (13.85)	0.493
Avoided/left the situation	74 (43.27)	53 (50.00)	21 (32.31)	0.023
Discussed with coworkers	129 (75.44)	79 (74.53)	50 (76.92)	0.724
Discussed with family/friends	26 (15.20)	19 (17.92)	7 (10.77)	0.206
Verbal report to supervisor	58 (33.92)	35 (33.02)	23 (35.38)	0.751
Written report to supervisor	13 (7.60)	8 (7.55)	5 (7.69)	0.972
Police/legal action	3 (1.76)	2 (1.90)	1 (1.54)	1.000
Workplace investigation				0.116
No investigation	96 (56.14)	53 (50.00)	43 (66.15)	
Investigation conducted	72 (42.11)	51(48.11)	21 (32.31)	
Not required	3 (1.75)	2 (1.89)	1 (1.54)	
Organizational response (Multiple responses)				
No action	40 (23.39)	26 (24.53)	14 (21.54)	0.654
Verbal warning	57 (33.33)	41 (38.68)	16 (24.62)	0.058
Written documentation	21 (12.28)	16 (15.09)	5 (7.69)	0.152
Disciplinary action	9 (5.26)	6 (5.66)	3 (4.62)	0.766
Legal action	2 (1.17)	1 (0.94)	1 (1.54)	1.000
Not required	3 (1.75)3	2 (1.89)	1 (1.54)	1.000
Reasons for not reporting (Multiple responses)				
Complex procedures	31 (18.13)	26 (24.53)	5 (7.69)	0.006
Lack of time	24 (14.04)	17 (16.04)	7 (10.77)	0.336
Lack of reporting knowledge	10 (5.85)	6 (5.66)	4 (6.15)	0.894
Fear of consequences	14 (8.19)	9 (8.49)	5 (7.69)	0.853
Perceived as useless	54 (31.58)	37 (34.91)	17 (26.15)	0.232
Feeling ashamed	2 (1.17)	1 (0.94)	1 (1.54)	1.000
Not required	11 (6.43)	6 (5.66)	5 (7.69)	0.526

Note: Differences in categorical variables on the chi-square test or Fisher’s exact test.

**Table 4:** Factors associated with WVP among nurses in healthcare settings in Thailand (*n* = 415).

Factors	Total (*n* = 415)		Urban (*n* = 205)	Rural (*n* = 210)
	Crude IRR (95% CI)	Adjusted IRR (95% CI)	Adjusted IRR (95% CI)	Adjusted IRR (95% CI)
Age (years)				
≥51	Reference	Reference	Reference	Reference
41–50	2.73 (1.11, 6.67) *	4.76 (1.52, 14.87) *	3.81 (0.98, 14.79)	9.13 (0.91, 91.14)
31–40	2.15 (0.85, 5.38)	3.08 (1.12, 8.47) *	2.88 (0.88, 9.42)	4.50 (0.53, 38.09)
21–30	3.23 (1.25, 8.36) *	3.31 (1.28, 8.56) *	3.07 (1.04, 9.07) *	4.06 (0.50, 32.87)
Sex				
Female	Reference	Reference	Reference	Reference
Male	1.29 (0.63, 2.62)	1.28 (0.63, 2.61)	1.42 (0.66, 3.07)	0.52 (0.07, 3.77)
Sexual minority	2.58 (0.96, 6.96)	2.61 (0.96, 7.07)	2.34 (0.73, 7.48)	2.50 (0.34, 18.14)
Marital Status				
Married	Reference	Reference	Reference	Reference
Single	1.25 (0.92, 1.71)	1.15 (0.81, 1.64)	1.32 (0.81, 2.15)	0.74 (0.42, 1.27)
Divorced/Separated	2.82 (1.47, 5.39) *	3.05 (1.54, 6.04) *	3.78 (1.66, 8.61) *	1.81 (0.40, 8.03)
Widowed	1.15 (0.15, 8.34)	2.55 (0.32, 19.73)	2.3 (0.28, 19.82)	-
Professional Nursing Experience				
Less than 1 year	Reference	Reference	Reference	Reference
1–5 years	1.23 (0.77, 1.97)	1.19 (0.74, 1.91)	1.60 (0.80, 3.17)	0.73 (0.37, 1.45)
6–10 years	1.20 (0.74, 1.95)	1.17 (0.72, 1.91)	1.67 (0.81, 3.45)	1.04 (0.50, 2.18)
11–15 years	1.10 (0.63, 1.94)	1.07 (0.59, 1.92)	2.17 (0.87, 5.38)	1.32 (0.46, 3.79)
16–20 years	1.20 (0.49, 2.97)	1.06 (0.41, 2.71)	1.91 (0.56, 6.44)	1.05 (0.11, 9.65)
20 years and above	1.10 (0.64, 1.90)	1.01 (0.56, 1.84)	2.38 (0.69, 8.16)	4.12 (0.82, 20.64)
Department of Work				
Private/VIP ward	Reference	Reference	Reference	Reference
ER	2.68 (1.22, 5.89) *	2.47 (1.11, 5.47) *	2.65 (0.78, 8.98)	1.59 (0.49, 5.10)
OPD	2.06 (0.88, 4.81)	2.11 (0.89, 5.01)	2.90 (0.79, 10.55)	1.17 (0.32, 4.24)
IPD	1.67 (0.76, 3.67)	1.48 (0.67, 3.29)	1.24 (0.35, 4.36)	1.49 (0.52, 4.29)
Medical ward	3.33 (1.60, 6.91) *	3.33 (1.60, 6.91) *	2.68 (0.83, 8.58)	3.24 (1.20, 8.71) *
Surgical ward	2.06 (0.94, 4.48)	2.10 (0.96, 4.58)	2.04 (0.61, 6.86)	1.57 (0.52, 4.70)
Pediatric ward	1.86 (0.81, 4.26)	1.92 (0.84, 4.38)	0.93 (0.20, 4.22)	2.49 (0.90, 6.87)
Other ^¥^	1.23 (0.50, 3.02)	1.22 (0.50, 3.01)	1.14 (0.25, 5.13)	1.23 (0.39, 3.80)
Shift/Work Schedule				
No shift work	Reference	Reference	Reference	Reference
Work in shifts	1.79 (0.95, 3.38)	1.83 (0.90, 3.71)	2.19 (0.95, 5.04)	1.37 (0.37, 5.06)
Work Hours				
Day only	Reference	Reference	Reference	Reference
Both day and night	1.80 (1.08, 3.00) *	2.03 (1.13, 3.64) *	2.51 (1.22, 5.15) *	1.23 (0.46, 3.29)
Type of Patients/Clients Served				
Mixed-patient groups	Reference	Reference	Reference	Reference
Male only	1.56 (1.09, 2.25) *	1.87 (1.28, 2.74) *	1.49 (0.95, 2.34)	1.65 (0.58, 4.68)
Female only	1.38 (0.97, 1.96)	1.54 (1.06, 2.22)	1.23 (0.76, 1.99)	1.77 (0.96, 3.27)
Pediatric only	1.44 (0.88, 2.37)	1.65 (0.99, 2.74)	0.78 (0.28, 2.17)	2.59 (1.39, 4.82)
Postpartum only	0.18 (0.02, 1.29)	0.20 (0.02, 1.49)	0.48 (0.06, 3.50)	-
Concern About Workplace Violence				
Not concerned	Reference	Reference	Reference	Reference
Slightly concerned	2.18 (1.13, 4.21) *	2.17 (1.12, 4.20) *	1.07 (0.42, 2.74)	3.67 (1.38, 9.75) *
Moderate concerned	2.64 (1.44, 4.86) *	2.51 (1.36, 4.63) *	1.72 (0.78, 3.80)	2.90 (1.11, 7.61) *
Very concerned	4.30 (2.32, 7.98) *	4.11 (2.21, 7.63) *	2.11 (0.94, 4.71)	6.80 (2.55, 18.09) *
Extremely concerned	4.87 (2.51, 9.46) *	4.57 (2.34, 8.93) *	2.76 (1.18, 6.42) *	4.69 (1.40, 15.69) *
Workplace Violence Reporting Procedure				
Yes	Reference	Reference	Reference	Reference
No	1.10 (1.10, 1.76) *	1.14 (0.71, 1.84)	1.09 (0.63, 1.89)	0.91 (0.33, 2.52)
Workplace Violence Reduction Policies				
Yes	Reference	Reference	Reference	Reference
No	1.30 (0.86, 1.96)	1.33 (0.88, 2.01)	1.40 (0.87, 2.26)	0.96 (0.41, 2.22)
Area				
Rural	Reference	Reference	-	-
Urban	1.69 (1.29, 2.21) *	1.68 (1.28, 2.20) *	-	-

* Statistically significant. ^¥^ Other departments include labor room, postpartum ward, gynecology, ENT, operating room, and other small units combined for analysis. WPV incidents over the past 12 months were analyzed using Poisson regression. Adjusted incidence rate ratios (IRRs) were calculated accounting for age, marital status, department of work, work hours, type of patients served, and level of concern about WPV. CI, confidence interval.

**Table 5:** Psychological responses following workplace violence among affected nurses by type of violence and setting (*n* = 181).

Psychological Response	Total	Urban	Rural	*p*-Value
	*n* (%)	*n* (%)	*n* (%)	
Physical violence (Multiple responses)	55 (30.39)	34 (18.78)	21 (11.60)	0.048
Hypervigilance/Heightened Alertness	42 (76.36)	27 (79.41)	15 (71.43)	0.498
Avoidance of Discussing the Incident	29 (52.73)	17 (50.00)	12 (57.14)	0.606
Recurrent Thoughts About the Incident	20 (36.36)	16 (47.06)	4 (19.05)	0.036
Reluctance to Come to Work	21 (38.18)	16 (47.06)	5 (23.81)	0.085
Intention to Resign or Transfer	20 (36.36)	17 (50.00)	3 (14.29)	0.007
Loss of Professional Confidence	21 (38.18)	15 (44.12)	6 (28.57)	0.249
Verbal abuse	171 (94.48)	106 (58.56)	65 (35.91)	<0.001
Hypervigilance or Heightened Alertness	102 (59.65)	65 (61.32)	37 (56.92)	0.569
Avoidance of Discussing the Incident	96 (56.14)	60 (56.60)	36 (55.38)	0.876
Recurrent Thoughts About the Incident	81 (47.37)	47 (44.34)	34 (52.31)	0.311
Reluctance to Come to Work	73 (42.69)	51 (48.11)	22 (33.85)	0.067
Intention to Resign or Transfer	74 (43.27)	54 (50.94)	20 (30.77)	0.010
Loss of Professional Confidence	70 (40.94)	51 (48.11)	19 (29.23)	0.015
Any violence	181 (43.61)	110 (53.66)	71 (33.81)	<0.001
Hypervigilance or Heightened Alertness	120 (66.30)	75 (68.18)	45 (63.38)	0.505
Avoidance of Discussing the Incident	100 (55.25)	62 (56.36)	38 (53.52)	0.707
Recurrent Thoughts About the Incident	86 (47.51)	52 (47.27)	34 (47.89)	0.936
Reluctance to Come to Work	76 (41.99)	53 (48.18)	23 (32.39)	0.036
Intention to Resign or Transfer	76 (41.99)	56 (50.91)	20 (28.17)	0.002
Loss of Professional Confidence	75 (41.44)	53 (48.18)	22 (30.99)	0.022

**Table 6:** Nurses’ recommendations for preventing workplace violence in healthcare settings by work setting (*n* = 415).

Suggested Preventive Measures	Total (*n* = 415)	Urban (*n* = 205)	Rural (*n* = 210)	*p*-Value
	*n* (%)	*n* (%)	*n* (%)	
Training on Violence Management	183 (44.10)	87 (42.44)	96 (45.71)	0.502
Increasing Staffing Levels	208 (50.12)	120 (58.54)	88 (41.90)	0.001
Improving Workplace Environment	189 (45.54)	111 (54.15)	78 (37.14)	0.001
Developing Violence Prevention Plans	185 (44.58)	97 (47.32)	88 (41.90)	0.267
Clear Disciplinary Measures	269 (64.82)	138 (67.32)	131 (62.38)	0.292
Strengthening Hospital Security Systems	211 (50.84)	115 (56.10)	96 (45.71)	0.034
Support from Organizational Leadership	146 (35.18)	90 (43.90)	56 (26.67)	<0.001
Effective Reporting Systems	130 (31.33)	68 (33.17)	62 (29.52)	0.423
Implementing Formal Policies	188 (45.30)	100 (48.78)	88 (41.90)	0.159

## Data Availability

The data supporting the findings of this study are not publicly available due to ethical and privacy considerations. Access to the data may be granted by the first author upon reasonable request and subject to approval by the relevant ethics committees and participating institutions.
